# Cell-Based Therapies for Heart Failure

**DOI:** 10.3389/fphar.2021.641116

**Published:** 2021-04-12

**Authors:** Antonio Carlos Campos de Carvalho, Tais H. Kasai-Brunswick, Adriana Bastos Carvalho

**Affiliations:** ^1^ Laboratory of Cellular and Molecular Cardiology, Institute of Biophysics Carlos Chagas Filho, Federal University of Rio de Janeiro, Rio de Janeiro, Brazil; ^2^ National Center of Structural Biology and Bioimaging (CENABIO), Federal University of Rio de Janeiro, Rio de Janeiro, Brazil; ^3^ National Institute of Science and Technology in Regenerative Medicine, Federal University of Rio de Janeiro, Rio de Janeiro, Brazil

**Keywords:** cell therapies, heart failure, bone marrow cells, adult stem cells, pluripotent cells (Min5–Max 8)

## Abstract

Heart failure has reached epidemic proportions with the advances in cardiovascular therapies for ischemic heart diseases and the progressive aging of the world population. Efficient pharmacological therapies are available for treating heart failure, but unfortunately, even with optimized therapy, prognosis is often poor. Their last therapeutic option is, therefore, a heart transplantation with limited organ supply and complications related to immunosuppression. In this setting, cell therapies have emerged as an alternative. Many clinical trials have now been performed using different cell types and injection routes. In this perspective, we will analyze the results of such trials and discuss future perspectives for cell therapies as an efficacious treatment of heart failure.

## Introduction

Cardiovascular diseases (CVD) are still the major cause of death in the world. The Global Burden of Disease (ghdx.healthdata.org) reports over 18.5 million deaths by CVD in the world in 2019, corresponding to 32.8% of all deaths. For comparison, in the years 1990 and 2000, these numbers were, respectively, 12 and 13.9 million deaths, corresponding to 25.9 and 27.5% of all deaths. People living with heart failure were estimated at 37.7 million worldwide in 2010 (Wollert et al., 2004) and are now estimated to have reached 63.3 million (AME Medical Journal, 2020). Mortality from HF is hard to estimate since the cause of death is usually attributed to the causative etiology, but 5-year survival rates for HF are similar to those of cancer and stroke (Askoxylakis et al., 2010).

The main etiologies for heart failure are ischemic, valvular, and hypertensive heart diseases, and primary and secondary cardiomyopathies. A detailed discussion of each etiology is beyond the scope of this perspective, and interested readers are referred to a review by Ziaeian and Fonarow (Ziaeian and Fonarow, 2016). According to the ESC guidelines (Ponikowski et al., 2016), heart failure is “a clinical syndrome characterized by typical symptoms (e.g., breathlessness, ankle swelling, and fatigue) that may be accompanied by signs (e.g., elevated jugular venous pressure, pulmonary crackles, and peripheral edema) caused by a structural and/or functional cardiac abnormality, resulting in a reduced cardiac output and/or elevated intracardiac pressures at rest or during stress.” The guidelines define a new term for heart failure (HF) in patients who have an ejection fraction (EF) between 40 and 49%: HF with midrange EF–HFmrEF. This new category now covers all ranges of EF in HF patients. HF with reduced EF (HFrEF) is defined as having ejection fractions ≤40% and HF with preserved EF (HFpEF) as ≥ 50%.

**FIGURE 1:**
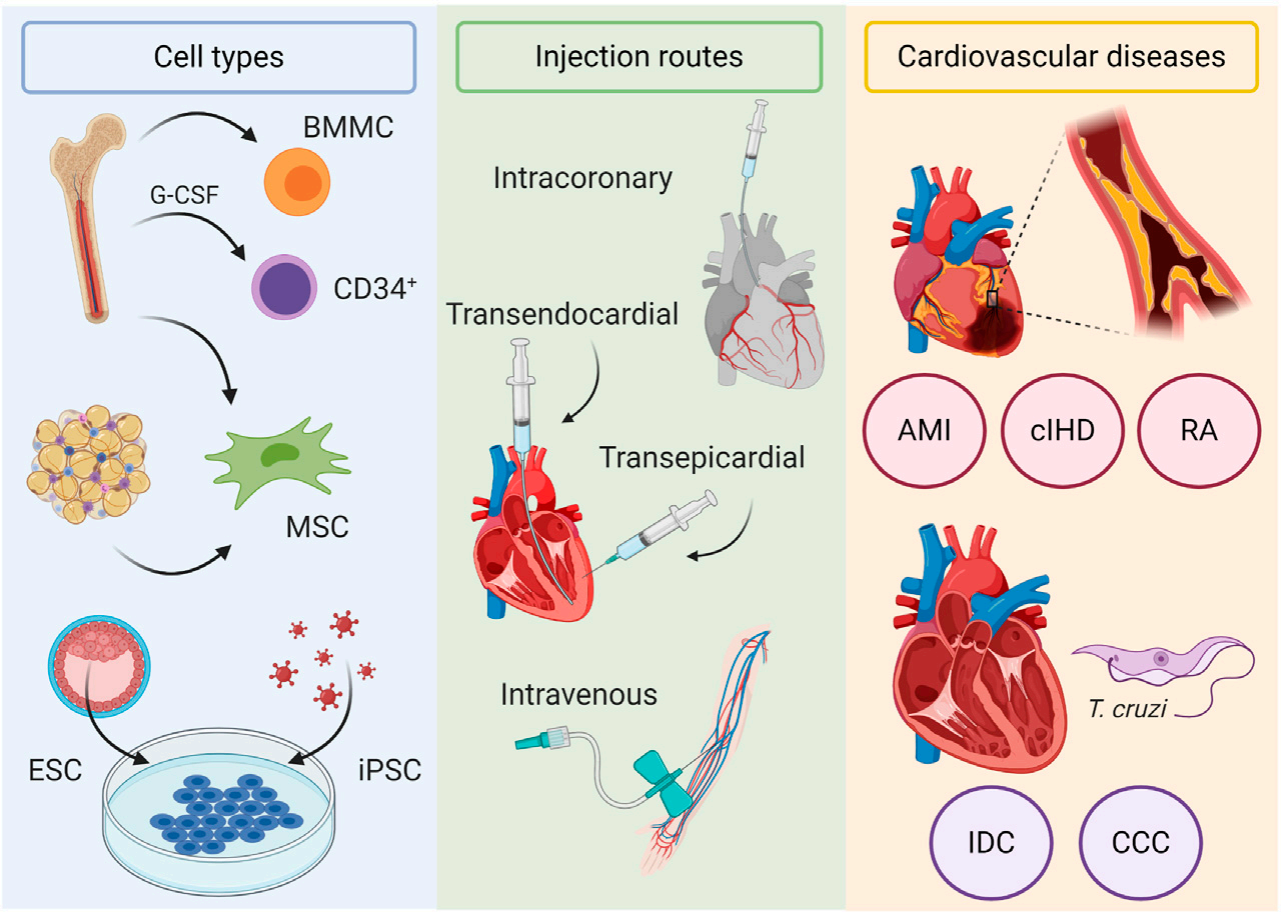
Cell-based therapies for heart failure. Cell types used in clinical trials include bone marrow mononuclear cells (BMMC), CD34^+^ mobilized with G-CSF, bone marrow and adipose tissue–derived mesenchymal stromal cells (MSC), embryonic stem cells (ESC), and induced pluripotent stem cells (iPSC). Injection routes include intracoronary, transendocardial, and transepicardial routes, which deliver cells directly in the myocardium, and intravenously. Cardiovascular diseases evaluated in clinical trials include ischemic (acute myocardial infarction (AMI), chronic ischemic heart disease (cIHD), and refractory angina (RA)) and nonischemic cardiomyopathies (idiopathic dilated cardiomyopathy (IDC) and chronic chagasic cardiomyopathy (CCC)).

Although there are many classes of pharmacologic drugs that are commonly used to treat HF and growing numbers of new molecules and signaling pathways are being investigated—for detailed information see (Yancy et al., 2016; Cresci et al., 2019), a number of HF patients do not respond adequately to optimal pharmacologic therapy and progress to congestive heart failure, when assist devices followed by heart transplantation are the only possible therapy. Donor organ shortage and complications related to continuous immunosuppression of these patients significantly limit this therapeutic option stimulating the search for new therapies. In this setting, cell-based therapies emerged as a potential treatment at the turn of the millennium. This perspective will review the clinical trials performed to date using different cell types and injection routes in distinct types of cardiac diseases that lead to heart failure (see Figure 1). Most of our analysis will focus on cell therapies for HFrEF and HFmrEF, where there is loss of cardiomyocytes due to various myocardial insults and cell-based therapies attempt to regenerate the lost cells.

## Cell-Based Therapies for Ischemic Heart Disease

Ischemic heart disease (IHD) is characterized by an unbalance between oxygen demand and supply, resulting in hypoxia that ultimately leads to cell death and loss of cardiomyocytes. Cell-based therapies were, therefore, initially proposed based on the assumption that the injected cell types could substitute or regenerate the lost cardiomyocytes. A pioneering attempt was the use of skeletal myoblasts by the group of Philippe Menasché in France (Menasché et al., 2001). At the turn of the millennium, research groups in Germany and Brazil started small clinical trials injecting autologous bone marrow-derived mononuclear cells (ABMMC) in patients that had suffered myocardial infarction either by intracoronary (Assmus et al., 2002; Strauer et al., 2002) or transendocardial (Perin et al., 2003; Gowdak et al., 2005) route. Results from these preliminary small trials suggested small functional cardiac improvements based on evaluation of left ventricular ejection fraction (LVEF) and indicated both procedures to be safe. Stimulated by these initial promising results, the number of clinical trials using the ABMMC significantly increased, and larger, more controlled trials were performed, supporting the initial positive results (Britten et al., 2003; Wollert et al., 2004; Schächinger et al., 2006a; Dill et al., 2009; Honold et al., 2012; Assmus and Zeiher, 2013). In 2006, two manuscripts, published back-to-back in the New England Journal of Medicine, reported discordant results on the effects of ABMMC in the setting of acute myocardial infarction (Lunde et al., 2006) (Schächinger et al., 2006b). During the following years more clinical trials using ABMMC in IHD were performed (Janssens et al., 2006; Grajek et al., 2010), as reported in meta-analysis studies (Lipinski et al., 2007; Martin-Rendon et al., 2008; Delewi et al., 2014; Assmus et al., 2015). For the rest of the decade, the prevailing view, although contested, was that bone-marrow-derived mononuclear cells were capable of some degree of heart repair after insult by IHD. In the following decade, at least ten randomized and multicenter trials using mostly ABMMC, but also selected bone marrow cells, in IHD occurred in the world. All of these trials did not find additional benefits to standard therapy (Traverse et al., 2011; Perin et al., 2012; Traverse et al., 2012; Sürder et al., 2013; Choudry et al., 2016; Sürder et al., 2016; Quyyumi et al., 2017; Wollert et al., 2017; Fernández-Avilés et al., 2018; Nicolau et al., 2018), mostly in acute but one in chronic IHD using either the intracoronary or the transendocardial injection route. Finally, the BAMI trial, a European effort to test the efficacy of ABMMC in acute myocardial infarction (AMI), did not meet planned endpoints relevant to clinical outcomes (mortality and MACE) between patients in the ABMMC and control groups due to low enrollment and low mortality (Mathur et al., 2020). As written in a thoughtful commentary by Roberto Bolli about the BAMI trial, cell therapy with bone marrow-derived mononuclear cells for AMI patients should “rest in peace” (Bolli, 2020).

But what about other types of IHD, such as chronic IHD (cIHD) and refractory angina? In chronic IHD, results are variable depending on cell type. Perin et al. have reported no benefit with a large ABMMC trial and positive results with a dose escalation study using high doses of allogenic bone marrow-derived mesenchymal stromal cells (MSC) in cIHD (Perin et al., 2012; Perin et al., 2015). Other studies performed by Hare’s group (Hare et al., 2012; Suncion et al., 2014) using autologous and allogeneic MSC injected transendocardially showed limited effects on global heart function in cIHD. A more robust study using a mixture of CD90^+^ MSC and CD45^+^/CD14^+^ bone marrow-derived cells delivered by transendocardial injection showed significant decreases in combined clinical outcomes that included composite all-cause death, cardiovascular hospital admissions, and unplanned clinical visits to treat decompensated heart failure (Patel et al., 2016). Two other studies using autologous bone marrow-derived MSC injected intramyocardially also found significant differences in surrogate endpoints such as reduction in left ventricular end systolic and diastolic volumes (LVSEV and LVEDV), but LVEF and myocardial mass did not vary significantly between cell and control groups (Teerlink et al., 2017) in one study, but reached statistical difference in the other (Mathiasen et al., 2015). It seems, therefore, that selected bone marrow cells directly injected in the myocardium may have a salutary effect in cIHD patients with heart failure. Nonetheless, most of these studies were phase 1-2 trials, and we should still wait for larger phase-3 trials.

Refractory angina is another type of IHD where cell-based therapies offer hope. In this setting the most important trials used selected CD34^+^ cells derived from the bone marrow. Positive results were consistently seen in phase 1-2 trials (Losordo et al., 2007, Losordo et al., 2011; Povsic et al., 2016), but the phase-3 trial had to be curtailed due to withdrawal of funding from industry (Henry et al., 2018). Since CD34 is a marker of endothelial progenitor cells (Asahara et al., 1997) the use of these cells to promote angiogenesis in the setting of IHD is rational, and unfortunately for the field of cell-based therapies the phase-3 clinical trial could not be completed, although pooled data from all the trials mentioned above showed significant increase in total exercise time, decreased angina frequency, and reduced 24-month mortality in patients receiving the cell therapy (Henry et al., 2018)

Other, non-marrow-derived cell types have also been tested in patients with IHD. Freshly isolated liposuction aspirate cells, MSC derived from adipose tissue, and putative cardiac stem/progenitor cells reached clinical studies but did not go beyond safety trials (Chugh et al., 2012; Makkar et al., 2012; Malliaras et al., 2014; Perin et al., 2014; Kastrup et al., 2017) for various reasons, including the disputed existence of a cardiac stem/progenitor cell (Berlo and Molkentin, 2014; Cai and Molkentin, 2017; Vicinanza et al., 2018; Aquila et al., 2019). The use of cells truly capable of generating new cardiomyocytes for heart repair, the pluripotent stem cells (either embryonic or induced pluripotent stem cells (iPSC)), waits further preclinical safety studies, although a small number of patients undergoing coronary artery bypass grafting (CABG) have received a fibrin patch of cardiac progenitor cells derived from human embryonic stem cells (Menasché et al., 2015, Menasché et al., 2018).

## Cell-Based Therapies for Nonischemic Cardiomyopathies

In the nonischemic cardiomyopathies, the major cause of the disease lies not in the blood supply to the heart but rather in the mechanical or electrical dysfunction of cardiomyocytes, although microvascular dysfunction and significant impairment in coronary blood flow reserve have been demonstrated in most patients with idiopathic dilated cardiomyopathy (Neglia et al., 2002; Canetti et al., 2003) . Cardiomyopathies can be classified as hypertrophic, dilated, restrictive, or arrhythmogenic. Cell-based therapies have been studied in two types of dilated cardiomyopathies: idiopathic dilated cardiomyopathy and chronic chagasic cardiomyopathy.

### Cell-Based Therapies for Idiopathic Dilated Cardiomyopathy

Since nonischemic dilated cardiomyopathies are one of the leading causes of advanced heart failure and account for more than 50% of heart transplants, cell-based therapies were also intensively investigated in this setting. There have been a number of small cell therapy trials in idiopathic dilated cardiomyopathy (IDC) using ABMMC that ascertained the safety and feasibility of the procedure (Seth et al., 2006; Fischer-Rasokat et al., 2009; Martino et al., 2010; Sant’Anna et al., 2014). Larger trials using ABMMC in IDC had conflicting results. Seth et al. (Seth et al., 2010) showed that, after 3 years, a total of 41 patients who received cells (versus 40 patients in the control arm) had a significant increase in LVEF and quality of life, although mortality did not differ between cell and control groups. On the other hand, Martino et al. (Martino et al., 2015) showed in 115 patients that completed the multicenter study that there were no significant differences in LVEF, quality of life, or mortality between groups. In both studies, cells were delivered by intracoronary injection but using slightly different methods. Other large trials in patients with IDC were performed using CD34^+^ cells derived from the bone marrow, delivered either by intracoronary or intramyocardial injection. When CD34^+^ were used, the cell-treated group showed increased LVEF, 6 min walking distance, decreased NT-proBNP levels and, surprisingly, given the small patient sample (55 in total), even a significant decrease in the combined secondary endpoint of 1-year mortality and heart transplantation (Vrtovec et al., 2011). A larger study enrolling 110 IDC patients followed for 5 years by the same group reported similar results (Vrtovec et al., 2013a). They further showed that injecting the CD34^+^ cells by the transendocardial route led to more cell retention and greater effects on LVEF, 6 min walking distance, and NT-proBNP levels than injecting by intracoronary route (Vrtovec et al., 2013b). MSC derived from bone marrow were also used in IDC patients. One trial using the same mix of CD90^+^ MSC and CD45^+^/CD14^+^ cells described above for ischemic heart disease (Patel et al., 2016) found that these cells did not improve clinical outcomes in nonischemic heart disease (Henry et al., 2014). A trial comparing the effects of autologous and allogeneic bone marrow MSC in IDC found greater, clinically meaningful efficacy with allogeneic cells (Hare et al., 2016), suggesting that these cells rather than the autologous should be tested in larger, more robust trials. In summary, the results of bone marrow-derived cell therapies in IDC seemed highly variable with the exception of the trials that used CD34^+^ selected cells, as was the case for the refractory angina patients. Since microvascular dysfunction and significant impairment in coronary blood flow reserve have been demonstrated in most patients with IDC (Neglia et al., 2002; Canetti et al., 2003), it is reasonable to assume that cells capable to promote angiogenesis, as CD34^+^ cells, could bring salutary effects to patients with IDC. Once again, we can only regret that larger, double-blinded, randomized, placebo-controlled, and multicenter trials have not been performed to definitely test the efficacy of these cells in IDC patients.

### Cell-Based Therapies for Chronic Chagasic Cardiomyopathy

Chronic chagasic cardiomyopathy (CCC) is a dilated cardiomyopathy caused by the parasite *Trypanosoma cruzi*, endemic in many regions in Latin America. Although the parasite, the transmitting vector, and the disease were described more than 100 years ago by Carlos Chagas (Chagas, 1909), disease pathogenesis mechanisms are still discussed, due to their multifactorial nature. A review of Chagas disease pathogenesis is beyond the scope of this perspective, but it is important to cite parasite persistence, autoimmunity, and microvascular alterations as pathogenic mechanisms (Tanowitz et al., 2009). Although bone marrow-derived cell therapies are not expected to eliminate parasites, their immunomodulatory and angiogenic capacities have been documented (Heldring et al., 2015). Furthermore, the use of bone marrow-derived cell therapies in CCC has been supported by innumerous preclinical model experiments (Soares et al., 2004; Goldenberg et al., 2008; Goldenberg et al., 2009; Soares et al., 2011; Jasmin et al., 2012; Jasmin et al., 2014; Iacobas et al., 2018). Based on promising results obtained in animal models of CCC treated with bone marrow-derived mononuclear cells, Vilas-Boas et al. conducted the first cell therapy trial in patients by intracoronary injection of ABMMC (Vilas-Boas et al., 2006; Vilas-Boas et al., 2011). This initial 28-patient trial proved the procedure to be safe and potentially efficacious, based on improvements in NYHA class, quality of life questionnaire, 6 min walking test, and LVEF. Based on that, a larger multicenter, randomized, placebo-controlled, and double-blinded trial was performed, evaluating 183 CCC patients (Santos et al., 2012). Results were disappointing since no statistical differences were observed between the cell-treated and placebo groups in all parameters listed above. To our knowledge no other clinical trials with cell therapies have been performed in CCC patients.

## Discussion

Clinical trials of cell-based therapies in heart failure have been performed using different cell types, cell doses, injection routes, and disease states. In addition, the majority of the trials performed recruited a small number of patients. For a comprehensive list of the clinical trials performed, see Supplementary Appendix A1 in the Appendix. All of these factors severely limited the conclusions about efficacy of these trials and the many, indeed excessive, meta-analyses performed did not take this profound heterogeneity into account. One clear result from all the clinical trials performed to date is that the procedures are safe and feasible, no matter what cell type or injection route is used for the attempted therapy of the distinct disease states.

The most commonly used cell type was undoubtedly a mixture of cells derived from the bone marrow, the mononuclear cell fraction, extracted directly by marrow aspiration or mobilized to the periphery by granulocyte colony stimulating factor (G-CSF). As mentioned above, the prevailing view, currently, is that the bone marrow mononuclear cell fraction does not bring additional benefit to patients, with either ischemic or nonischemic heart disease, over conventional pharmacologic therapy. The last, long waited BAMI trial could not be completed and has put to rest any hopes that these cells might impact clinically meaningful endpoints. Other cell types are still waiting the final verdict, but with the exception of the CD34^+^ cells (limited to angiogenesis) it is unlikely that any other marrow-derived cell type may bring real benefits to heart failure patients. In our opinion, hope lies in pluripotent stem cells, the only ones capable of differentiation into any cell type. In fact, robust engraftment and muscularization have been shown with human embryonic and induced pluripotent stem cell-derived cardiomyocytes in AMI in nonhuman primates (Chong et al., 2014; Shiba et al., 2016; Liu et al., 2018), although transient arrhythmias have been recorded and further preclinical safety studies are therefore required. Nonetheless, a small number of patients undergoing coronary artery bypass grafting (CABG) have received a fibrin patch of cardiac progenitor cells derived from human embryonic stem cells (Menasché et al., 2015, Menasché et al., 2018) without serious adverse events being recorded. Injection routes have varied extensively among the clinical trials performed. The two most used routes were the intracoronary and the intramyocardial, the last one either by endocardial or epicardial access. In a small number of trials, intravenous injection of cells has also been attempted (Butler et al., 2016). The intramyocardial route seems to be more effective in clinical trials that compared it to intracoronary delivery of the cells (Brunskill et al., 2009; Vrtovec et al., 2013b; Mozid et al., 2014). At any rate, cell delivery and survival in clinical trials have been very limited independent of the injection route chosen (Hofmann et al., 2005; Fonseca et al., 2011; Spoel et al., 2011).

Cell doses have also varied among clinical trials but have usually been in the range of 10^7^–10^9^ cells either using unselected or selected bone marrow-derived cells (Sanganalmath and Bolli, 2013). A few studies have compared different cell doses and found that lower cell doses were more efficient than the higher doses (Hamamoto et al., 2009; Hare et al., 2012).

In summary, almost more than 20 years after the first clinical trials of cell-based therapies for heart disease were started, the field has yet to demonstrate a robust result that would allow cell therapies to be incorporated into clinical practice. Throughout this period, it became evident that unselected bone marrow cells are not an adequate cell source. Mesenchymal stromal cells offer limited improvement and, in our opinion, will not reach clinical practice. CD34^+^ cells seem to work for refractory angina, but the efficacy trial was never completed. We envisage that when the transient arrhythmias induced by cardiomyocytes derived from pluripotent stem cells in the nonhuman primates are better understood and controlled, these cells will be capable to enter clinical trials and hopefully allow cardiologists to offer a viable option for heart failure patients who are candidates for assist devices and heart transplantation.

## Data Availability

The original contributions presented in the study are included in the article/Supplementary Material; further inquiries can be directed to the corresponding authors.
