# Supplementary material for: Cell-Based Therapies for Heart Failure
Source: Front Pharmacol. 2021 Apr 12;12:641116. doi: 10.3389/fphar.2021.641116 (PMC8072383; doi:10.3389/fphar.2021.641116)
Supplement: Supplementary file 1 [file datasheet1.docx]

| **ACUTE MYOCARDIAL INFARCTION – AMI** | | | | | | | |
| --- | --- | --- | --- | --- | --- | --- | --- |
| **Cell type and orign** | **Cell number (x10^6^)** | **Injection route** | **Clinical trial info** | **N**  **(cell *vs* control)** | **Endpoint** | **Reported results** | **Ref** |
|  |  |  |  |  |  |  |  |
| Autologous  BMMC  Autologous  CBPC | 245  NI | IC | \| Single center  Randomized  Open-label \| \| --- \| \|  \| | 9 (9:0)  11 (11:0) | LVEF | Positive | Assmus et al. 2002 |
| Autologous  BMMC | 28 | IC | Single center  Randomized  Placebo-controlled | 20 (10:10) | LVEF | Positive | Strauer *et al.*2002 |
| Autologous  BMMC | 2460 ± 940 | IC | Randomized Placebo -controlled | 60 (30:30) | LVEF | Positive | Wollert et al.2004 |
| Autologous  BMMC | 236 ± 174 | IC | Phase III Multicenter Randomized Placebo-controlled | 204 (101:103) | LVEF | Positive | Schachinger *et al. 2006*  Dill *et al.2008* |
| Autologous  BMMC | 68 | IC | Multicenter  Randomized | 100 (50:50) | LVEF | no effect | Lunde *et al.*2006 |
| Autologous  BMMC | 304 ± 128 | IC | Multicenter  Randomized  Placebo-controlled | 67 (33: 34) | LVEF | no effect | Janssens *et al.*  2006 |
| Autologous BMMC | 2340 ± 1200 | IC | Single center Randomized | 45 (31: 14) | LVEF | no effect | Grajek *et al.* 2010 |
| Autologous BMMC | 50-500 | IC | Phase II Multicenter Randomized Open labeled | 150 (95: 55) | LVEF | no effect | Surder *et al.* 2013 2016 |
| Autologous BMMC | 150 | IC | Multicenter Randomized Placebo- controlled | 67(43: 24)  day 3  53 (36: 17)  day 7 | LVEF | no effect | Traverse *et al.* 2012 |
| Autologous BMMC | 150 | IC | Multicenter Randomized Placebo-controlled | 87 (58: 29) | LVEF | no effect | Traverse *et al.* 2011 |
| Autologous BMMC | 59.8 ± 59.9 | IC | Phase II Multicenter Randomized Placebo - controlled | 98 (54: 44) | LVEF | no effect | Choudry *et al.* 2015 |
| Autologous BM CD34+ | ≥ 10 | IC | Phase II Multicenter Randomized Placebo – controlled | 161 (78: 83) | LVEF | no effect | Quyyumi *et al.* 2017 |
| Autologous BMMC | 2000 ± 700 | IC | Multicenter Randomized Placebo -controlled | 50 (38: 12)  low dose  47 (33: 14)  high dose  25 (25: 0)  low irradiated  31 (31: 0)  high irradiated | LVEF | no effect | Wollert *et al.* 2017 |
| Allogenic  CSC | 10; 20; 35 | IC | Multicenter Randomized Placebo – controlled | 49( 33: 16) | Safety | Safe | Fernández-Avilés *et al.* 2018 |
| Autologous BMMC | 100 | IC | Multicenter Randomized Placebo -controlled | 121 (66: 55) | LVEF | no effect | Nicolau *et al.* 2018 |
| Autologous BMMC | 25-500 | IC | Multicenter Randomized Open-label | 375 (185: 190) | Mortality  MACE | Inconclusive | Marthur *et al.* 2020 |
| **CHRONIC ISCHEMIC HEART DISEASE – cIHD** | | | | | | | |
| Autologous BMMC | 25.5 ± 6.3 | TE | Single center Randomized Placebo -controlled | 21 (14: 7) | LVEF | Positive | Perin *et al.* 2003 |
| Autologous  G-CSF CPC | 29 | IC | Phase I Single center Randomized | 32 (23: 9) | Global and regional LV functional  LV Volumes | Positive | Honold *et al.* 2012 |
| Autologous BMMC | 100 | TE | Phase II Single center Randomized Placebo – controlled | 92 (61: 31) | Myocardial perfusion LVESV Max. oxygen consumption | no effect | Perin *et al.* 2012 |
| Autologous and Allogeneic BM-MSC | 20  100  200 | TE | Phase I/II Multicenter Dose-escalation study Randomized Open-label | 10:0  10:0  10:0 | Safety Remodelling | Positive | Hare *et al. 2012* Suncion et al.2014 |
| Allogeneic BM-MSC | 25  75  150 | TE | Phase II Multicenter Dose-escalation  Randomized Placebo-controlled | 20 (15: 5)  20 (15: 5)  20 (15: 5) | Safety  MACE | no effect | Perin *et al.* 2015 |
| Autologous BM-MSC | NI | IM | Multicenter Randomized Placebo-controlled | 55 (37: 18) | LVESV | Positive | Mathiasen *et al.* 2015 |
| Autologous Ixmyelocel-T | NI | TE | Phase IIb Multicenter Randomized Placebo - controlled | 226 (160: 66) | All-cause death, Cardiovascular admission to hospital, Unplanned clinic visits to treat acute decompensated heart failure | Positive | Patel *et al.* 2016 |
| Autologous BM-MSC | ≥ 600 | IM | Multicenter Randomized Placebo -controlled | 315 (157: 158) | Remodelling | Positive | Teerlink *et al.* 2017 |
| Autologous CSC c-kit | 1 or 0.5 | IC | Phase I Randomized Open-label | 33 (20: 13) | Safety   LV function | Positive | Chugh *et al.* 2012 |
| Autologous CDC | 12.5 | IC | Phase I Multicenter Dose-escalation Randomized Open-label | 25 (17: 8) | Safety | Positive | Makkar *et al.* 2012 |
| Autologous ADRC | 0.4; 0.8; 1.2 (cells/kg) | TE | Phase I Multicenter Randomized Placebo - controlled | 27 (21: 6) | Safety | Positive | Perin *et al.* 2014 |
| Autologous CDC | 12.5 - 25 | IC | Phase I Randomized Placebo -controlled | 25 (17: 8) | Safety | Positive | Malliaras *et al.* 2014 |
| Autologous Ixmyelocel-T | 35 to 295 | mTC | Phase II Multicenter Randomized SOC | 30 (21: 9) | Safety  MACE | Positive | Henry *et al.*2014 |
| Autologous CDC | 25 | IC | Phase I/II Multicenter Randomized Double-blind Placebo - controlled | 69 (49: 20) (recent MI)  65 (45: 20) (chronic MI) | Safety Infarct size | Positive | Chakravarty *et al. 2017* |
| Autologous Sca-1+-ESC | 4 | EDP | Case report | 1 (1: 0) | Immunologic reaction  LV function | Safe | Menasché *et al.* 2015 |
| Autologous Sca-1+-ESC | 8.2 | EDP | Phase I Single center Open label | 6 (6: 0) | Safety | Positive | Menasché *et al.* 2018 |
| **Refractory Angina** | | | | | | | |
|  |  |  |  |  |  |  |  |
| Autologous BMMC | 21.5 | IM | Case report | 1 (1: 0) | Safe | Positive | Gowdak *et al.* 2005 |
| Autologous BM CD34+  G-CSF mobilized | - 1. (cells/kg)   0.5 (cells/kg) | IM | Phase II Multicenter Randomized  Placebo-controlled | 78 (53: 25)  78 (53: 25) | Weekly angina frequency | Positive | Losordo *et al.* 2017 |
| Autologous BM CD34+  G-CSF mobilized | 0.1 or 0.5 (cells/kg) | IM | Phase II Multicenter Randomized Placebo - controlled | 78 (50: 28) | Safety  Frequency of angina symptoms | Interrupted | Povsic *et al.* 2016 |
| Autologous BM CD34+  G-CSF mobilized | 0.01 or 0.1 or 0.5  (cells/kg) | IM | Phase III Multicenter Randomized Double-blind Placebo - controlled  Open label  SOC | 304 (187: 89 28 SOC) | Total exercise time Frequency of angina symptoms All-cause mortality | Interrupted | Henry *et al.* 2018 |
| **IDIOPATHIC DILATED CARDIOMYOPATHY – IDC** | | | | | | | |
|  |  |  |  |  |  |  |  |
| Autologous BMMC | 259 ± 135 | IC | Phase I Single center Open-label | 33 (33: 0) | Absolute change in regional LV wall motion of the target area | Positive | Fischer-Rasokat *et al.*2009 |
| Autologous BMMC | 176-295 | IC | Phase I Multicenter Randomized Placebo -controlled | 115 (61: 54) | LVEF | no effect | Martino *et al.2*015 |
| Autologous BMMC | 28 ± 16 (cells/ mL) | IC | Phase I Randomized Open-label | 44 (24: 20) | NYHA functional class LEVF Mortality | Positive | Seth *et al.*2006 |
| Autologous BMMC | 168 ± 96 | IC | Phase I Randomized Open-label | 81 (41: 40) | NYHA  KCCQ LEVF Mortality | Positive | Seth *et al.*2010 |
| Autologous BMMC | 106 | mTC | Phase I Randomized Open-label | 30 (20: 10) | LEVF | no effect | Sant`anna *et al.*2014 |
| Autologous PB-GCSF  CD34+ | 123 ± 23 | IC | Phase I Randomized Open-label | 55 (28: 27) | LVEF NT-proBNP | Positive | Vrtovec *et al.*2011 |
| Autologous PB-GCSF  CD34+ | 103 ± 27  105 ± 31 | IC  TE | Phase I Randomized Open label | 20 (20: 0)  20 (20: 0) | LVEF | TE had better results  than IC | Vrtovec *et al.*2013 |
| Autologous PB-GCSF  CD34+ | 113 ± 26 | IC | Phase I Randomized Open label | 110 (55: 55) | LVEF | Positive | Vrtotec *et al.*2013 |
| Autologous Ixmyelocel-T | 35 to 295 | IM | Phase II Multicenter Randomized | 29 (18: 11) | Safety  MACE | no effect | Henry *et al.*2014 |
| Autologous MSC Allogeneic MSC | 100 | TE | Single center Randomized | 16 (16: 0)  18 (18:0) | Safety  MACE NYHA class | Positive  (allogenic) | Hare *et al.*2016 |

AMI: acute myocardial infarct; cIHD: chronic ischaemic heart disease; IDC: idiophatic dilated cardiomyopathy; BMMC: bone marrow mononuclear cells; CBPC: circulating blood progenitor cells; G-CSF: granulocyte colony-stimulating factor; CPC: circulating progenitor cells; BM-CD34+: CD34+ cells derived from bone marrow; G-CSF: granulocyte colony-stimulating factor; CSC: cardiac stem cell; BM-MPC: bone marrow mesenchymal precursor cells; MSC: mesenchymal stem cell; ixmyelocel-T: mixture of bone marrow CD90+ cells and CD45+/CD14+ cells; CSC c-kit+: cardiac stem cell c-kit+; CDC: cardiac-derived cell; Sca-1+ ESC: Embryonic stem cell - cardiac progenitor Sca-1+ cells; PB-GCSF CD34+: peripheral blood- GCSF mobilized D34+ cells; IC: intracoronary; TE: transendocardial; mTC: mini-thoracotomy; EDP: epicardically-derived patch; SOC: standard of care; LVEF: left ventricular ejection fraction; LV: left ventricule; LVESV: left ventricular end systolic volume; MACE: major adverse cardiac effects; NI: not indicated; NYHA: New York Heart Association; KCCQ: Kansas City Cardiomyopathy Questionnaire; NT- proBNP: N-terminal (NT)-pro hormone BNP; NI: not indicated.
